# Tissue Nonspecific Alkaline Phosphatase Function in Bone and Muscle Progenitor Cells: Control of Mitochondrial Respiration and ATP Production

**DOI:** 10.3390/ijms22031140

**Published:** 2021-01-24

**Authors:** Zhi Zhang, Hwa Kyung Nam, Spencer Crouch, Nan E. Hatch

**Affiliations:** 1 Department of Natural Sciences, University of Michigan-Dearborn, 4901 Evergreen Rd, Dearborn, MI 48128, USA; zhizhan@umich.edu; 2 School of Dentistry, University of Michigan-Ann Arbor, 1011 N University Avenue, Ann Arbor, MI 48103, USA; hknam@umich.edu (H.K.N.); crouchsp@umich.edu (S.C.)

**Keywords:** tissue nonspecific alkaline phosphatase, mitochondria, bone marrow stromal cell, bone, muscle, nucleotide adenosine triphosphate

## Abstract

Tissue nonspecific alkaline phosphatase (TNAP/*Alpl*) is associated with cell stemness; however, the function of TNAP in mesenchymal progenitor cells remains largely unknown. In this study, we aimed to establish an essential role for TNAP in bone and muscle progenitor cells. We investigated the impact of TNAP deficiency on bone formation, mineralization, and differentiation of bone marrow stromal cells. We also pursued studies of proliferation, mitochondrial function and ATP levels in TNAP deficient bone and muscle progenitor cells. We find that TNAP deficiency decreases trabecular bone volume fraction and trabeculation in addition to decreased mineralization. We also find that *Alpl*^−/−^ mice (global TNAP knockout mice) exhibit muscle and motor coordination deficiencies similar to those found in individuals with hypophosphatasia (TNAP deficiency). Subsequent studies demonstrate diminished proliferation, with mitochondrial hyperfunction and increased ATP levels in TNAP deficient bone and muscle progenitor cells, plus intracellular expression of TNAP in TNAP+ cranial osteoprogenitors, bone marrow stromal cells, and skeletal muscle progenitor cells. Together, our results indicate that TNAP functions inside bone and muscle progenitor cells to influence mitochondrial respiration and ATP production. Future studies are required to establish mechanisms by which TNAP influences mitochondrial function and determine if modulation of TNAP can alter mitochondrial respiration in vivo.

## 1. Introduction

Tissue nonspecific alkaline phosphatase (TNAP) is a cellular enzyme that is encoded by the *Alpl* gene. TNAP is well known for its role in tissue mineralization, in which TNAP functions as an ectoenzyme in cell and matrix vesicle membranes to hydrolyze pyrophosphate to inorganic phosphate in the extracellular space [1]. Because pyrophosphate is a strong inhibitor of mineralization [2] while phosphate is a substrate for mineralization, TNAP activity promotes collagenous matrix mineralization [3,4]. In fact, TNAP is essential for bone mineralization such that lack of adequate TNAP leads to the metabolic disorder hypophosphatasia [5,6]. More severe and earlier onset forms of hypophosphatasia have predominant signs of poor bone mineralization with symptoms of bone bending, pain and fracture leading to mobility limitations, respiratory issues due to weak ribs, muscle weakness and fatigue, as well as seizures and even death [7]. Later onset and milder forms of hypophosphatasia (HPP) also show signs of poor bone mineralization that over time can lead to pain and fracture with limited bone remaining for good surgical repair [8,9]. Fortunately, through the diligent work of multiple investigators, individuals with severe hypophosphatasia can now be treated with enzyme replacement therapy using a bone targeted recombinant form of TNAP enzyme [10,11,12]. This drug (Strensiq^®^, Alexion Pharma GmbH, Zurich, Switzerland), has proven extremely successful at improving survival, bone mineralization, bone strength, and mobility in the treated individuals [13]; and is now considered the treatment of choice for the earlier onset more severe forms of this metabolic disorder [14].

While the function of TNAP in tissue mineralization is widely accepted, it is important to recognize that TNAP plays additional important roles in progenitor cells during development, including a function in cell stemness in multiple tissue types [15,16,17,18,19]. Relevant to skeletal development, TNAP is expressed in bone rudiments several days prior to ossification [20,21] and has also been identified as a marker of multipotent bone marrow stromal stem cell subpopulations [22]. TNAP positive progenitor cells retain the ability to differentiate into osteogenic, adipogenic and chondrogenic lineages, while TNAP negative cells do not [22]. In addition, expression of TNAP in bone marrow decreases with age and TNAP deficiency has been implicated in mesenchymal cell senescence leading to bone ageing [23].

Based upon these results, plus the fact that our prior studies demonstrated that TNAP is essential for control of cell cycle, proliferation and differentiation of cranial bone progenitor cells [24]; here, we sought to determine if TNAP is needed in a cell autogenesis manner for osteogenesis using a subcutaneous implant model of collagen mixed with bone marrow stromal cells isolated from *Alpl*^+/+^ or *Alpl*^−/−^ mice. We also extended our prior findings from bone to muscle, and demonstrate for the first time that *Alpl*^−/−^ mice (TNAP global knockout mice) exhibit a phenotype that includes muscle weakness and diminished motor coordination, similar to that seen in individuals with infantile hypophosphatasia. At the cellular level, we show that TNAP is expressed in bone and muscle progenitor cells in an intracellular pattern, and that TNAP modulates mitochondrial function and ATP levels in these cells. Such results shed new insight into mechanisms by which TNAP may function in bone and muscle progenitor cells to influence musculoskeletal health. Our results may also be relevant to metabolic disorders, given that mitochondrial dysfunction and TNAP are associated with obesity, insulin resistance, and metabolic syndrome [25,26].

## 2. Results

### 2.1. TNAP Deficiency Decreases Bone Mineralization and Trabecular Bone Formation by Bone Marrow Stromal Cells

**Figure 1:**
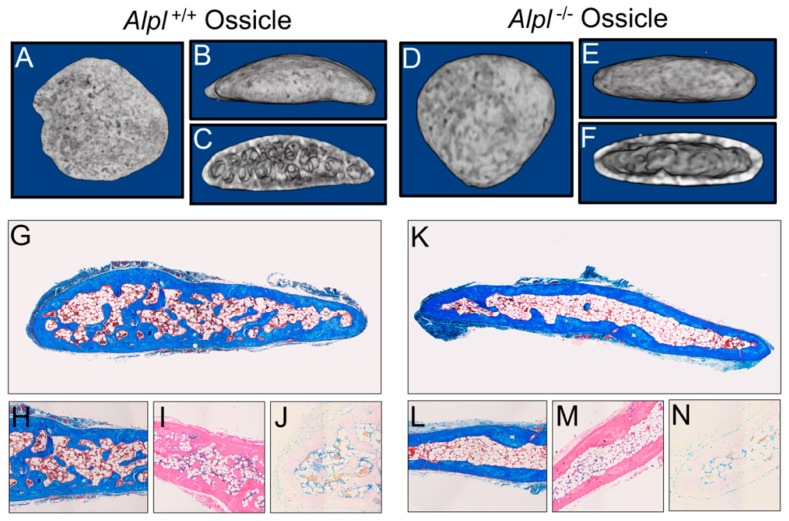
Representative nano CT isosurface renderings and histology of *Alpl*^+/+^ and *Alpl*^−/−^ ossicles after eight weeks of implantation. (**A**–**F**), Nano CT isosurface renderings of *Alpl*^+/+^ (**A**–**C**) and *Alpl^−/−^* (**D**–**F**) ossicles after 8 weeks of implantation. (**G**–**N**), Histologic stains of *Alpl*^+/+^ (**G**–**J**) and *Alpl*^−/−^ (**K**–**N**) ossicles after 8 weeks of implantation. (**G**–**J**), *Alpl*^+/+^ ossicles and (**K**–**N**), *Alpl*^−/−^ ossicles. Mason’s trichrome (**G**,**H**,**K**,**L**); hematoxylin and eosin (**I**,**M**); alkaline phosphatase (**J**,**N**) stains. Note lack of bone trabeculae in *Alpl*^−/−^ ossicles as seen by histology and isosurface images.

Representative nano CT isosurface renderings show that the bone marrow stromal cell (BMSC) collagen implants (ossicles) from both *Alpl*^+/+^ and *Alpl*^−/−^ mice formed a cortical bone shell after 8 weeks of subcutaneous implantation. However, decreased trabeculation was apparent in the *Alpl*^−/−^ as compared to *Alpl*^+/+^ ossicles (Figure 1A–F). Representative histologic stains confirm decreased trabecular bone evident in *Alpl^−/−^* when compared to the *Alpl^+/+^* ossicles (Figure 1G–N). As expected, alkaline phosphatase staining (purple) was decreased in the *Alpl^−/−^* as compared to *Alpl^+/+^* ossicles (Figure 1J,N). It is worth noting that, while the represented *Alpl^−/−^* ossicle stain appears to show increased adipocytes compared to the represented *Alpl^+/+^* ossicle stain, we did not find consistent differences in adipocity of ossicles between the two genotypes.

**Figure 2:**
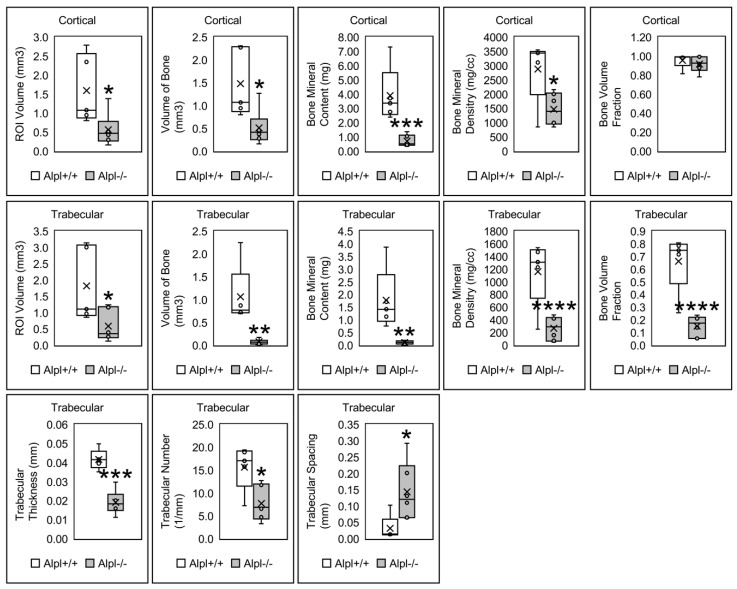
Cortical and trabecular bone parameters of *Alpl*^+/+^ and *Alpl*^−/−^ ossicles. Results shown are means ± standard error. *n* = 5 per genotype. cort = cortical; trab = trabecular; * *p* value < 0.05 between genotypes; ** *p* value < 0.01 between genotypes; *** *p* value < 0.005 between genotypes, **** *p* value < 0.001.

Quantitative nano computed tomography (nano CT) of ossicle bone supports the representative images shown in Figure 1 and Figure 2. Cortical bone parameters demonstrate a significant decrease in bone mineralization indices (bone mineral content and bone mineral density) in BMSC implants from *Alpl*^−/−^ mice. Non-normalized cortical bone volume was decreased in *Alpl*^−/−^ mice; however, there was no significant difference in the bone volume fraction between *Alpl*^−/−^ and *Alpl*^+/+^ mice. Therefore, the cortical bone volume was dependent upon the size of the ossicle (ossicles with *Alpl*^+/+^ cells were larger than those with *Alpl*^−/−^ cells), not the genotype of the BMSCs. In contrast, trabecular bone parameters demonstrate a significant decrease in bone mineralization indices (bone mineral content, bone mineral density) in implants from *Alpl*^−/−^ mice in addition to decreased indices of trabecular bone volume fraction, trabecular thickness, and trabecular number in *Alpl*^−/−^ ossicles. Concordantly, trabecular separation is increased in *Alpl*^−/−^ as compared to *Alpl*^+/+^ ossicles.

We also performed micro computed tomography (micro CT) on tibia bones from the *Alpl*^−/−^ and *Alpl*^+/+^ donor mice (Supplementary Figure S1). Results are consistent with the nano CT results from BMSC ossicle implants in that cortical bone volume when normalized to total volume was not significant while parameters of bone mineralization (bone mineral content, bone mineral density) were significantly diminished in tibias from *Alpl*^−/−^ as compared to *Alpl*^+/+^ donor mice. Also similar to the nano CT analyses of the ossicles, measures of trabecular bone including trabecular bone volume fraction (bone volume/total volume), trabecular number, trabecular thickness were all significantly decreased, while trabecular spacing was significantly increased, in tibias from *Alpl*^−/−^ as compared to *Alpl*^+/+^ donor mice. Representative images of alizarin and Alcian blue stained tibia shows the degree of mineralization defects in long bones of *Alpl*^−/−^ as compared to *Alpl*^+/+^ donor mice (Supplementary Figure S2).

### 2.2. TNAP Deficiency Increases BMSC Total and Adipocyte Colony Forming Units

**Figure 3:**
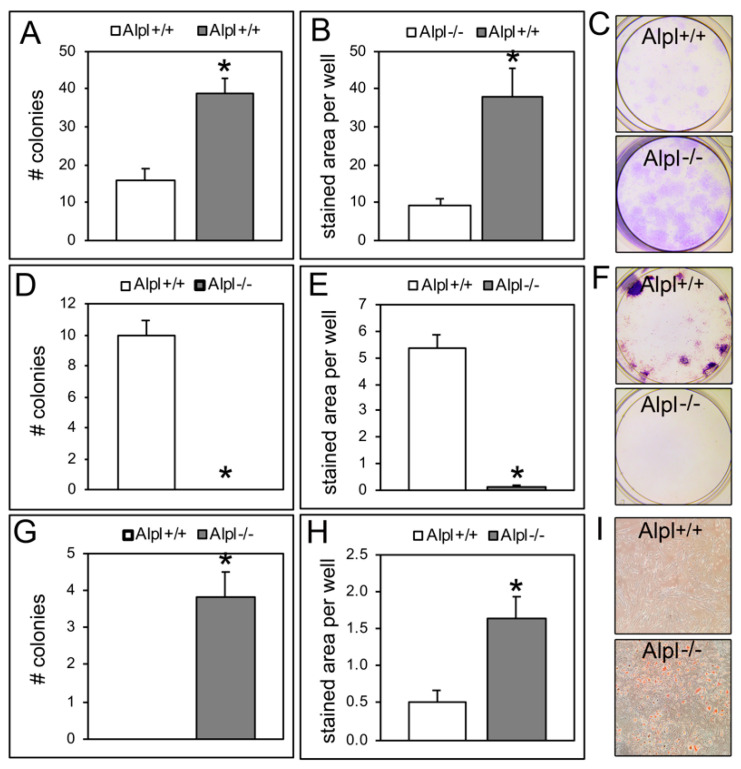
*Alpl*^−/−^ bone marrow stromal cells exhibit increased total colony forming units and adipocyte colony forming units. (**A**) The total number of crystal violet-stained colonies was significantly increased in *Alpl^−/−^* compared to *Alpl*^+/+^ BMSCs (bone marrow stromal cells). (**B**) The area of crystal violet stain per well significantly increased in *Alpl^−/−^* compared to *Alpl*^+/+^ BMSCs. (**C**) Representative images of crystal violet stain in *Alpl^+/+^* BMSCs and *Alpl^−/−^* BMSCs. (**D**) The total number of colonies stained for alkaline phosphatase significantly decreased in *Alpl^−/−^* compared to *Alpl*^+/+^ BMSCs. (**E**) The area of alkaline phosphatase stain per well significantly decreased in *Alpl^−/−^* BMSCs. (**F**) Representative images of alkaline phosphatase stain in *Alpl^+/+^* BMSCs and *Alpl^−/−^* BMSCs. (**G**) The total number of colonies stained with oil red (for adipocytes) significantly increased in *Alpl^−/−^* as compared to *Alpl*^+/+^ BMSCs. (**H**) The area of oil red stain per well significantly increased in *Alpl^−/−^* BMSCs. (**I**) Representative images of oil red stain in *Alpl^+/+^* BMSCs and *Alpl^−/−^* BMSCs. *n* = 3 per genotype. * *p* < 0.05, statistical significance between genotypes. # = number.

*Alpl*^−/−^ BMSCs formed more overall colonies as stained with crystal violet than *Alpl*^+/+^ cells when cultured immediately after isolation from host animals (passage 0 cells) (Figure 3A–C). As expected, *Alpl*^−/−^ cells formed no alkaline phosphatase positive colonies while *Alpl*^+/+^ cells did form alkaline phosphatase positive colonies (Figure 3D–F). *Alpl*^−/−^ cells also formed significantly more adipocyte colonies as stained with oil red when compared to *Alpl*^+/+^ cells (Figure 3G–I).

### 2.3. TNAP Deficiency Increases Both Osteoblast and Adipocyte Differentiation of BMSCs

**Figure 4:**
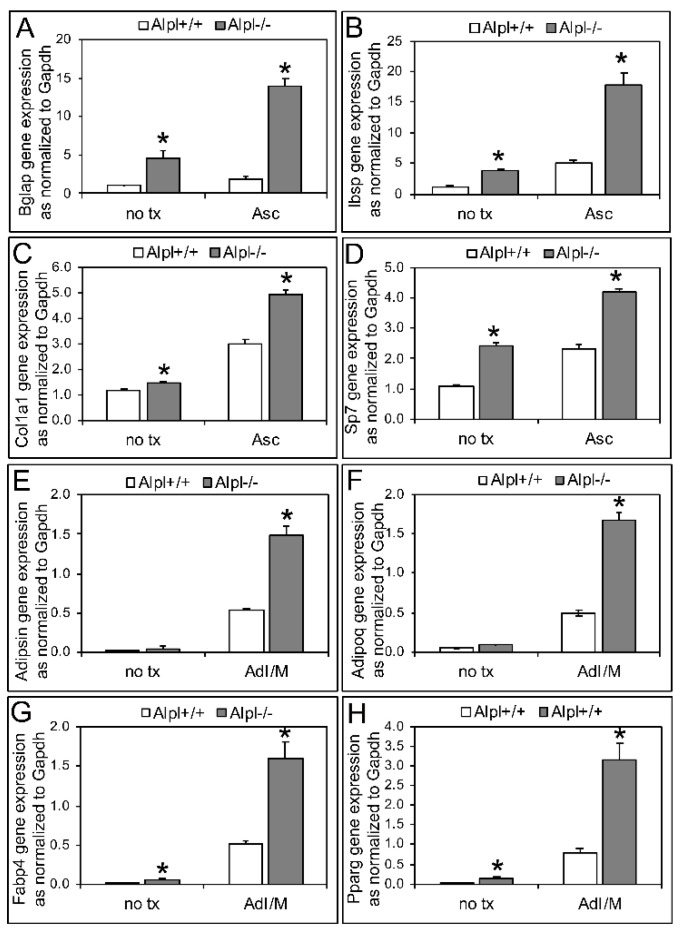
TNAP deficiency increases osteoblastic and adipocytic differentiation in *Alpl^−/−^* bone marrow stromal cells (BMSCs) as assessed by gene expression. The osteoblast genes, *Bgla*p (**A**), *Ibsp* (**B**), *Col1a1* (**C**), and *Sp7* (**D**) significantly increased in the *Alpl^−/−^* compared to *Alpl^+/+^* BMSCs, when cultured in non-differentiation media (no tx) or osteoblast differentiation media containing ascorbate (Asc) for 6 days. The adipocyte genes, *Adipsin* (**E**), *Adipoq* (**F**), *Fabp4* (**G**), and *Pparg* (**H**) significantly increased in the *Alpl^−/−^* compared to *Alpl^+/+^* BMSCs, when cultured in adipocyte induction then maintenance media (AdI/M) for 6 days total. Passage 2 cells were used. *n* = 3 per genotype. * *p* < 0.05, statistical significance between genotypes.

Despite lack of alkaline phosphatase positive *Alpl*^−/−^ colony formation, analysis of mRNA revealed that *Alpl*^−/−^ cells expressed higher levels of osteoblast genes (*Bgla*p, *Ibsp*, *Col1a1*, *Sp7*) compared to *Alpl*^+/+^ cells when cultured in non-differentiation media or media containing ascorbate to induce osteoblast differentiation (Figure 4A,C,E,G). Consistent with the colony forming units (CFU) oil red staining results, *Alpl*^−/−^ cells also expressed higher levels of adipocyte genes (*Pparg, Fabp4*, *Adipsin*, *Adipoq*) compared to *Alpl*^+/+^ cells when cultured in adipocyte induction and maintenance media (Figure 4B,D,F,H).

### 2.4. TNAP Deficiency Decreases Muscle Strength and Impairs Motor Coordination

**Figure 5:**
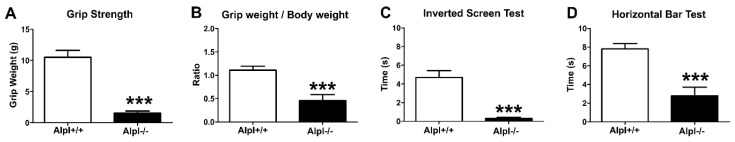
TNAP deficiency decreases muscle strength and impairs motor coordination. Muscle strength and motor coordination tests were carried out in *Alpl*^+/+^ (*n* = 11) and *Alpl*^−/−^ (*n* = 13) littermates at 14 days old. (**A**) Grip strength is significantly decreased in *Alpl*^−/−^ as compared to *Alpl*^+/+^ mice. (**B**) The ratio of grip strength/body weight is significantly decreased in *Alpl*^−/−^ as compared to *Alpl*^+/+^ mice. (**C**) *Alpl*^−/−^ mice fell off the inverted screen significantly earlier as compared to *Alpl*^+/+^ mice. (**D**) *Alpl*^−/−^ mice fell off the horizontal bar significantly earlier as compared to *Alpl*^+/+^ mice. *** *p* < 0.001, statistical significance between genotypes.

Because individuals with hypophosphatasia exhibit muscle weakness in addition to motor coordination deficiencies, we assessed *Alpl*^−/−^ mice for muscle strength and coordination as compared to wild type littermates (Figure 5). Grip strength and grip strength normalized for body weight were significantly decreased in *Alpl*^−/−^ mice, compared to their *Alpl*^+/+^ littermates. In addition, *Alpl*^−/−^ mice fell off the inverted screen and horizontal bar much earlier, as compared to the *Alpl*^+/+^ mice.

### 2.5. TNAP Deficiency Diminishes Progenitor Cell Proliferation and Increases Cell Metabolic Activity

**Figure 6:**
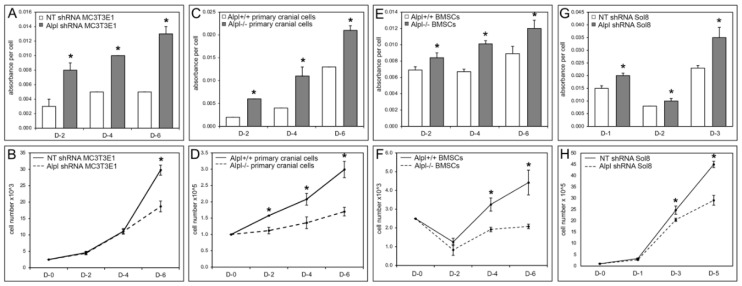
TNAP deficiency increases progenitor cell metabolic activity concurrent with decreased progenitor cell proliferation. (**A**) Cell metabolic activity (as measured by reduction of the tetrazolium dye, MTT) is significantly increased in *Alpl* shRNA treated MC3T3E1 cranial osteoprogenitors at days 2, 4, and 6 after plating, as compared with non-target control shRNA treated MC3T3E1 cells. (**B**) The number of cells is significantly decreased in *Alpl* shRNA MC3T3E1 cranial osteoprogenitors at days 2, 4, and 6 after plating, as compared with non-target shRNA MC3T3E1 cells. Similar results (increased metabolic activity with decreased proliferation) were also found for *Alpl*^−/−^ compared to *Alpl*^+/+^ primary cranial cells (**C**,**D**); *Alpl*^−/−^ compared to *Alpl*^+/+^ bone marrow stromal cells (BMSCs) (**E**,**F**); and *Alpl* shRNA as compared to non-target shRNA Sol8 skeletal muscle progenitor cells (**G**,**H**). *n* = 3 per genotype. * *p* < 0.05, statistical significance between genotypes. The Sol8 cell growth experimental period is shorter due to higher proliferation rate of these, as compared to the other cell types. Note: standard deviations were very low in some groups such that, while present, the bars are not visually apparent on the graphs (**A**,**C**,**E**,**G**).

Abnormalities in mitochondrial cristae were previously reported in muscle biopsies of a sheep model of hypophosphatasia [27]. Mitochondrial dysfunction could account for the diminished strength and motor skills of individuals and mice with severe hypophosphatasia [14,28]. As an initial step towards determining if TNAP influences mitochondrial function, we measured cell metabolic activity as evidenced by reduction of the tetrazolium dye, MTT, in cranial bone progenitor cells (primary cranial cells and MC3T3E1 cells), bone marrow stromal cells (BMSCs) and muscle progenitor cells (Sol8 cells). Because reduction levels of MTT can be influenced by cell number, we also assessed cell proliferation in concurrent experiments. Cell metabolic activity was significantly increased in MC3T3E1 cranial osteoprogenitors transduced with *Alpl* shRNA as compared to control non-target shRNA, in *Alpl*^−/−^ as compared to *Alpl*^+/+^ primary cranial osteoprogenitors, in *Alpl*^−/−^ as compared to *Alpl*^+/+^ bone marrow stromal cells, and in Sol8 muscle progenitor cells transduced with *Alpl* shRNA as compared to non-target shRNA (Figure 6A,C,E,G). All TNAP deficient cell types concurrently showed significantly diminished cell proliferation when compared to control cells (Figure 6B,D,F,H).

### 2.6. TNAP Deficiency Alters Mitochondrial Activity and Cell Respiration

**Figure 7:**
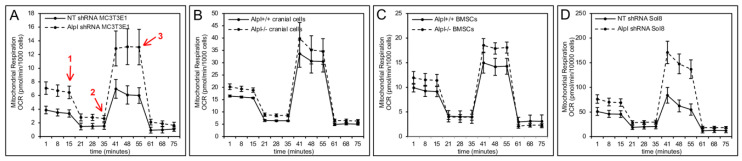
TNAP deficiency increases mitochondrial respiration and oxygen consumption rate. Seahorse XF cell mito stress tests (Agilent) were performed to confirm an effect of TNAP on live cell metabolic activity. Red arrows demark when cells are exposed to respiration modulators. 1 = oligomycin (inhibits ATP synthase; reflects loss of ATP linked respiration); 2 = FCCP (disrupts the mitochondrial membrane potential; reflects maximal respiration); 3 = Rotenone + Antimycin (inhibit mitochondrial proton pumps; reflect nonmitochondrial respiration driven by processes outside the mitochondria). Minutes 1–15 reflect basal cellular oxygen consumption rate (OCR). Minutes 15–35 reflect loss of ATP linked OCR. Minutes 41–55 reflect maximal OCR. Basal and maximal OCR appear increased in in *Alpl* shRNA treated vs. non-target shRNA MC3T3E1 cells (**A**), in *Alpl*^−/−^ vs. *Alpl*^+/+^ primary cranial osteoprogenitor cells (**B**), in *Alpl*^−/−^ vs. *Alpl*^+/+^ BMSCs (**C**), and in *Alpl* shRNA treated vs. non-target shRNA Sol8 skeletal progenitor cells (**D**). *n* = 3 per genotype per experiment. Statistical comparisons for key parameters of mitochondrial function are shown in Table 1.

Oxygen consumption rate (OCR) reflects the rate of mitochondrial and non-mitochondrial respiration, and can be measured in live cells with/without specific mitochondrial electron transport chain modulators, to evaluate and measure mitochondrial function. Results on live cells over time appear to demonstrate differences in mitochondrial function upon TNAP deficiency in MC3T3E1 cranial osteoprogenitors, primary cranial bone osteoprogenitors, bone marrow stromal cells and Sol8 muscle progenitor cells (Figure 7). Statistical comparisons from these assays demonstrate that all tested TNAP deficient progenitor cells exhibit significantly increased levels of basal respiration and ATP production (Table 1). Both mitochondrial proton leak and maximal respiration levels were significantly increased in TNAP deficient MC3T3E1 cells, primary cranial osteoprogenitors, and Sol8 muscle progenitors, but not in TNAP deficient bone marrow stromal cells. Space respiratory capacity (measures how closely cells are respiring relative to maximal respiratory ability) and non-mitochondrial enzyme activity were also significantly increased in the Sol8 skeletal muscle progenitor cell line.

### 2.7. TNAP Deficiency Increases Intracellular ATP Levels in Bone Marrow Stromal and Sol8 Muscle Progenitor Cells

**Figure 8:**
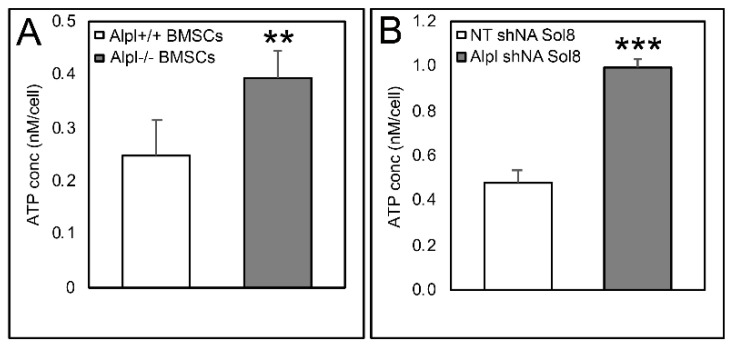
TNAP deficiency increases intracellular ATP levels. The intracellular ATP concentration significantly increased in *Alpl^−/−^* BMSCs (**A**), and *Alpl* shRNA treated Sol8 cells (**B**). *n* = 3 per genotype per experiment. ** *p* < 0.01, *** *p* < 0.001, statistical significance between genotypes.

We next measured ATP in cells to confirm results established by the live cell mitochondrial metabolic function assay described above. Results confirm that intracellular ATP levels were significantly increased in *Alpl*^−/−^ as compared to *Alpl*^+/+^ bone marrow stromal cells and in Sol8 muscle progenitor cells that expressed *Alpl* shRNA as compared to non-target shRNA (Figure 8).

### 2.8. TNAP is Localized Internally and Co-Localizes with Mitochondria

**Figure 9:**
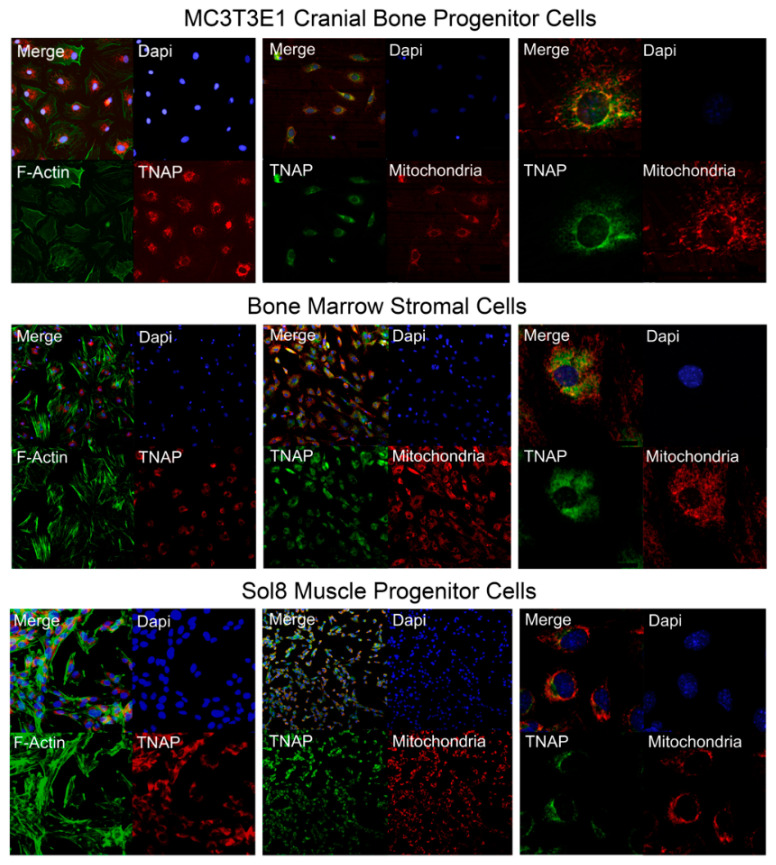
TNAP is expressed in a peri-nuclear pattern and partially co-localizes with mitochondria in undifferentiated bone and muscle progenitor cells. Immunofluorescent staining of undifferentiated MC3T3E1 cranial osteoprogenitor cells, bone marrow stromal cells, and Sol8 muscle progenitor cells reveals that TNAP is localized internally at a peri-nuclear region, and is co-localized with mitochondria. Left panels: representative images of co-localization of TNAP (red) and F-Actin (green). Middle panels: representative images of co-localization of TNAP (green) and mitochondria (red). Right panels: representative images of co-localization of TNAP (green) and mitochondria (red) at a higher magnification. Nuclear stain was performed with DAPI (blue).

Our results indicate that TNAP alters mitochondrial function and ATP production in bone and muscle progenitor cells. Therefore, we next sought to determine where TNAP is localized within these cells. Immunofluorescent staining for F-Actin (a cytoskeletal component) in combination with staining for TNAP demonstrates that TNAP is expressed in a peri-nuclear intracellular pattern in undifferentiated MC3T3E1 cranial bone progenitor cells, bone marrow stromal cells, and Sol8 muscle progenitor cells (Figure 9). To determine the spatial relationship between TNAP and mitochondria, we next co-stained for TNAP and mitochondria. Results show that both mitochondria and TNAP are located in a peri-nuclear pattern around the nucleus (Figure 9). Co-localization of TNAP and mitochondria occurs to different degrees in the three cells types; in many cells TNAP localizes near but not in the same place as mitochondria (Figure 8). It is worth noting here that the intracellular location of TNAP was confirmed through use of two different primary antibodies (Abcam ab65834 and R&D MAB29091).

## 3. Discussion

In this study, we first investigated bone marrow stromal cells isolated from *Alpl^−/−^* mice, to determine if lack of TNAP expression causes cell autonomous defects in osteogenesis and/or differentiation of these bone progenitor cells. To test this hypothesis in a 3D matrix in vivo, we mixed bone marrow stromal cells (BMSCs) from either *Alpl^−/−^* and *Alpl^+/+^* mice with a collagen carrier, then implanted them subcutaneously in immunodeficient donor mice for eight weeks to allow for bone formation. We found that *Alpl^−/−^* cells successfully formed a bone cortical shell similar in bone volume fraction but decreased in mineralization, when compared to those of *Alpl^+/+^* cells. This finding is consistent with the widely accepted and well proved concept that TNAP is essential for bone mineralization. Notably, we also found that implants of *Alpl^−/−^* cells had significantly less trabecular bone volume fraction and trabeculation in addition to reduced mineralization, when compared to those of *Alpl^+/+^* cells. If the role of TNAP is solely to facilitate matrix mineralization, we would have found trabecular bone that was of equal volume and trabeculation with decreased mineralization. Micro CT of tibial bones from *Alpl^−/−^* and *Alpl^+/+^* donor mice confirmed the results of the collagenous implants, in that trabecular bone volume fraction and trabeculation were significantly diminished in tibias of *Alpl^−/−^* mice. These results are consistent with previously published studies. For example, in a prior study, bone volume fraction and trabecular thickness in addition to bone mineral density, were significantly reduced in tibias of *Alpl*^−/−^ mice as compared to wild type littermates [29]. Together, these finding demonstrate a need for TNAP in BMSCs for trabecular osteogenesis that extends beyond that of matrix mineralization. Such findings are consistent with our prior studies which showed that cranial bone progenitor cells have a cell autonomous need for TNAP that influences cell cycle progression, cytokinesis, and proliferation [24]. TNAP deficiency may therefore decrease the pool of osteoprogenitors needed for bone formation in certain bone cell populations. Additional studies are required to definitively state that this is the case. Our results also suggest that TNAP may have differential impacts on cortical and trabecular bone formation.

To better understand how TNAP influences BMSCs, we next performed colony forming assays using isolated cells that were not previously passaged. We were surprised to find that *Alpl^−/−^* cells formed more colony forming units (CFUs) when stained with crystal violet than *Alpl^+/+^* cells. This data is inconsistent with previous studies which showed that human TNAP positive BMSCs had significantly more colony forming units than TNAP negative BMSCs [22]. It is likely that the increased crystal violet stained CFUs are not caused by increased proliferation of *Alp^l^*^−/−^ cells because the subsequent proliferation data shows decreased BMSC proliferation. Decreased proliferation was also present in other tested TNAP deficient cells including cranial osteoprogenitors and muscle progenitors. It is worth noting that crystal violet staining of CFUs reflects the number of viable cells from an isolation that result in adherent bone marrow cells. We interpret the increase in crystal violet-stained colonies of *Alpl^−/−^* BMSCs to reflects an increased adherence of those cells during the culturing process and/or an increase in cell size of these cells, which we previously found in TNAP deficient cranial osteoprogenitor cells [24]. Increased adherence and/or increase cell size of TNAP deficient cells could account for the increased crystal violet-stained colony forming unit assay results in the presence of diminished proliferation.

As expected, *Alpl^−/−^* cells did not form alkaline phosphatase positive colonies, while *Alpl^+/+^* cells did. This is easily explained by the lack of TNAP activity in the *Alpl^−/−^* cells. To determine if the *Alpl^−/−^*BMSC’s were diminished in their overall ability to differentiate osteoblasts, we performed real time PCR which demonstrated increased expression of osteoblast differentiation markers both prior to and during differentiation. This indicates that *Alpl^−/−^* BMSCs are in fact more disposed to osteoblast differentiate.

*Alpl^−/−^* cells also formed more adipocyte colony forming units as stained with oil red, compared to *Alpl^+/+^* cells. To confirm this result, we performed real time PCR which demonstrated increased expression of adipocyte associated genes and transcription factors. The findings of increased adipogenesis and expression of adipocyte differentiation markers in *Alpl^−/−^* cells are consistent with prior studies which demonstrated that alkaline phosphatase negative mesenchymal subpopulations retain adipocytic potential [30]. It is possible that *Alpl^−/−^* cells did not form trabecular bone in the collagenous implants because *Alpl^−/−^* cells had a greater tendency to form adipocytes than osteoblasts though the fact that *Alpl^−/−^* cells also showed increased osteoblast mRNA expression suggests that TNAP is more likely essential for the overall BMSC proliferation vs. differentiation fate switch.

It is important to note that while results from this study show that TNAP deficiency decreases trabecular bone volume and promotes bone marrow stromal cell osteoblast and adipocyte differentiation, while decreasing proliferation, we interpret our results to indicate that TNAP is essential for trabecular bone osteogenesis. This is based upon prior studies of *Alpl*^−/−^ mice that showed significant differences in osteoblast but not osteoclast function between *Alpl*^−/−^ and *Alpl*^+/+^ mice [31]. A limitation of this study is that we did not study osteoclastogenesis or function in *Alpl*^−/−^ implanted ossicles or tibia.

Because HPP patients have diminished muscular strength and fatigue issues, we tested muscle strength and motor coordination in *Alpl^+/+^* and *Alpl^−/−^* mice littermates. We found that *Alpl^−/−^* mice phenocopy these deficiencies seen in individuals with TNAP deficiency [14,28], as indicated by significantly decreased grip strength and grip strength normalized to body weight, in addition to significantly diminished endurance and motor coordination in inverted screen and horizontal bar tests. Because the *Alpl^−/−^* mice exhibit decreased muscle strength and coordination, we created Sol8 skeletal muscle cells that stably express *Alpl* shRNA to include TNAP deficient muscle progenitor cells in our subsequent studies.

In this study, we used the reduction of MTT assay followed by live cell *Seahorse* assay to establish that TNAP deficiency leads to defects in mitochondrial activity and cell respiration in bone and muscle progenitor cells. We found that cellular respiration and ATP production were significantly higher in all of the four tested TNAP-deficient bone and muscle progenitor cell types. The increase in ATP was confirmed in subsequent direct measurements of intracellular ATP in BMSCs and TNAP deficient Sol8 muscle progenitor cells. Extracellular ATP levels were also significantly increased in the media of the TNAP deficient cultured cells (data not shown), but extracellular levels were approximately 500× less than that found intracellularly. Notably, while not directed compared, baseline levels of ATP in *Alpl*^+/+^ cells were higher in the Sol8 muscle progenitor cells than the bone marrow stromal cells. The increase in ATP found in *Alpl*^−/−^ cells was also greater in Sol8 muscle progenitor than bone marrow stromal cells. Sol8 cells also showed less variation in ATP measurements as compared to bone marrow stromal cells. Together, this indicates that TNAP may have a greater effect on ATP production in muscle compared to bone progenitor cells.

The idea that TNAP influences mitochondrial function and ATP levels is not new. The upregulation of TNAP in vascular smooth muscle cells causes mitochondrial dysfunction and diminishes ATP intra- and extracellular levels [32]. In addition, mitochondrial cristae abnormalities were previously reported in a sheep model of hypophosphatasia [27]. While not directly investigated in this study, prior results indicate potential mechanisms by which mitochondrial hyperfunction and ATP levels can influence progenitor cells. Mitochondrial hyperfunction can promote cell senescence (irreversible loss of cell proliferation) and/or apoptosis in BMSCs and other cell types [23,33]. In BMSCs, evidence indicates that TNAP deficiency induced increases in intracellular ATP contribute to senescence of BMSCs via repression of the AMPKα pathway [23]. Increased mitochondrial oxidative phosphorylation function can also promote BMSC differentiation as mediated by ATP or b-catenin [34].

Mitochondria also regulate many critical cellular processes in skeletal muscles, including muscle cell metabolism, energy supply, and calcium homeostasis [35]. In this study, *Alpl* shRNA treated Sol8 muscle progenitor cells showed mitochondrial hyperfunction, including increased basal respiration and ATP production. The continuously increased basal mitochondrial respiration in TNAP deficiency may therefore induce chronic oxidative stress, which can result in superoxide production and induce muscle pathological changes [36]. Optimized mitochondrial function, including control of generated reactive oxidative species and quality control of mitochondrial proteins, are essential for maintaining muscle mass and muscle function [35]. In addition, the high levels of oxidative phosphorylation seen in TNAP deficient cells should increase inner mitochondrial membrane potential, potentially driving calcium into the inner mitochondrial space from calcium stores such as the sarcoplasmic reticulum [37]. Low calcium levels in the sarcoplasmic reticulum could therefore contribute to muscle weakness. High ATP levels seen in TNAP deficient cells would also be expected to decrease activation of AMPK, which could in turn lead to diminished glucose uptake and muscle activity [38]. Given that we investigated TNAP function in cultured Sol8 muscle progenitor cells, our results indicate a direct influence of TNAP on muscle progenitor cells that is likely mediated by mitochondrial changes. TNAP is also known to influence neural progenitor cells, development, and function [39,40]; therefore, it is also possible that TNAP deficiency causes loss of muscle strength, increased fatigue, and loss of motor coordination due to TNAP deficiency in neurons. TNAP regulates purinergic transmission in the central nervous system, and plays an important role in neuronal development, differentiation, and synaptic function [41]. TNAP inhibition also increases extracellular ATP in neurons, which can reduce motoneuron neurite extension [42], and dysregulate synaptic transmission in neuromuscular junction [43]. In addition, TNAP deficiency leads to reduced brain white matter, accompanied by decreased axonal myelination in the spinal cord and cortex [44]. Therefore, it is possible that TNAP deficiency induced high ATP levels influence muscle function directly, and/or via deficient neural function, the latter of which would impair neural control of muscle and result in decreased muscle function.

The relationship between TNAP deficiency and mitochondrial function in our and others prior studies made us question the widely held belief that TNAP is only expressed on extracellular membranes. Accordingly, we next used immunostaining to detect TNAP in bone and muscle progenitor cell lines. Here we report, for the first time, that TNAP is expressed in a peri-nuclear intracellular pattern in bone and muscle progenitor cells. Because this is a novel finding, we used two different primary antibodies for TNAP to confirm these results. Intracellular location of TNAP in progenitor cells calls into question the use of fluorescent-activated cell sorting (FACS) for isolation of TNAP positive/negative from bone marrow stromal and/or other cell populations, because this type of sorting will miss those cell that express TNAP intracellularly. On a positive note, the knowledge that TNAP can be expressed inside and/or outside the cell may help to reconcile the contradictory results showing influences of TNAP on bone marrow stromal and other cell populations [15,16,22,30,45,46,47,48]. We also found that TNAP co-localizes in part with mitochondria. In future studies it will be important to determine if TNAP is tethered to mitochondrial membranes, and/or to intracellular membranes adjacent to mitochondria, and further delineate how TNAP influences mitochondrial function. Notably, it was previously shown that high inorganic pyrophosphate levels prevent mitochondrial membrane depolarization in ischemic cardiac muscle cells [49]. We are currently pursuing studies of TNAP and Enpp1 (enzymatic hydrolyzer of pyrophosphate) double knockout mice to determine if the high pyrophosphate levels present in TNAP deficient (*Alpl*^−/−^) mice mediates the mitochondrial respiration changes seen in cells from these mice.

Results of this study are also pertinent to studies of TNAP and mitochondria in metabolic syndrome. High serum alkaline phosphatase levels are associated with higher risk of metabolic syndrome in the U.S. population [26]. Mechanisms between this association are unknown. Insulin resistance, obesity, and/or metabolic syndrome can induce mitochondrial dysfunction and mitochondrial ultrastructural abnormalities, which can be passed down through at least three generations via the female germline [25,50,51,52,53,54]. While much discussion in the literature has suggested that mitochondrial dysfunction linked to the pathogenesis and/or progression of metabolic disease can be explained by downstream effects of mitochondria generated reactive oxygen species, our results indicate that TNAP itself may influence mitochondrial function, which could be relevant to the worldwide epidemics of obesity, diabetes, and metabolic syndrome.

## 4. Materials and Methods

### 4.1. Animals

Wild type (*Alpl^+/+^*) and TNAP knockout (*Alpl*^−/−^) mouse littermates were bred on a 97% 129/SVJ (Jackson Laboratory, Bar Harbor, ME, USA) and 3% C57BL/6 (Charles River Laboratory, Wilmington, MA, USA) genetic background. This transgenic mouse model of infantile hypophosphatasia (HPP) represents the more severe phenotype of infantile HPP in the human population. Because TNAP is essential for vitamin B6 metabolism [55], all of the mice in this study were given free access to modified laboratory rodent diet containing 325 pyridoxine. Genotyping was performed by PCR using DNA samples from tail digests. *Alpl*^+/+^ primers (TGCTGCTCCACTCACGTCGAT and ATCTACCAGGGGTGCTAACC) and *Alpl^−/−^* primers (GAGCTCTTCCAGGTGTGTGG and CAAGACCGACCTGTCCGGTG) were used as previously described [3,56]. Six-week-old male CB17-SCID immunocompromised mice were obtained from Charles River Laboratory (Charles River Laboratory, Wilmington, MA, USA) and used as recipient mice for subcutaneous implant experiments. All animal procedures were performed according to U.S.A. federal guidelines and the Declaration of Helsinki. Prior to experimentation, animal protocols were approved by the Institutional Animal Care and Use Committee (IACUC) of the University of Michigan (protocol PRO00008675, expiration 1/2/2022).

### 4.2. Bone Marrow Stromal Cell Isolation

Bone marrow stromal cells (BMSCs) were isolated from femurs of 14-day-old *Alpl^−/−^* mice and wild type (*Alpl^+/+^*) littermates. Briefly, epiphyseal growth plates were removed and the marrow was collected by flushing with a 25-gauge needle and a 5 mL syringe containing media. Marrow cell clumps were aspirated several times through a 22-gauge needle and filtered through a 70-µm cell strainer. Cells were cultured in a custom formulated αMEM containing no ascorbate, supplemented with 20% fetal bovine serum (FBS), penicillin/streptomycin (P/S), and fungizone for several days. Media were changed every 12 h for 2 days, then every 3 days until all suspension cells were removed and adherent cells were confluent. Significantly greater numbers of cells were isolated from *Alpl^+/+^* (2.2 × 10^6^ ± 2.2 × 10^5^ BMSCs per mouse) than *Alpl^−/−^* mice (1.1 × 10^6^ ± 1.6 × 10^5^ per mouse, *p* < 0.05). During bone marrow aspiration, long bones of the *Alpl^−/−^* mice were noticeably obstructed with hard tissue in the mid-diaphyseal region such that diminished cell numbers isolated from these mice were likely due to diminished overall mouse/bone size and diminished bone marrow per bone.

### 4.3. Collagenous Implant Preparation

Collagenous gel implants were prepared using isolated BMSCs. Cells of passage 3 were used for fabrication of implants. 2 × 10^6^ bone marrow stromal cells per implant were suspended with 0.01% NaOH in phosphate buffered saline (PBS) and 4 µg/µL of rat tail collagen type I (Corning, Tewksbury, NY, USA) on ice. The solution was then aliquoted into glass tissue chamber slide wells (Thermo Fisher Scientific, Waltham, MA, USA) for total gel volumes of 200 µL per implant and final collagen gel concentration of 3.0 mg/mL. Gel solutions with cells were incubated at 37 °C for 1 h to enable gel hardening.

### 4.4. Subcutaneous Implant Placement and Nano Computed Tomography of Ossicles

Midline longitudinal incisions were made along the dorsal surface of each host mouse and subcutaneous pockets were formed by blunt dissection. A single implant was placed into each subcutaneous pocket, for a total of two implants per animal. Each mouse received two *Alpl*^−/−^ BMSC implants, two *Alpl*^+/+^ BMSC implants, or two blank (no cells) collagenous implants. Implants were removed eight weeks after implantation. After fixation, implants were analyzed for mineralized tissue formation by nano computed tomography (nano CT) scanning (Phoenix Nanotom M nano computed tomography imaging system and associated software, GE Healthcare Pre-Clinical Imaging, London, ON, Canada) at a 9 µm isotropic voxel resolution. Implants were then decalcified and embedded in paraffin for histologic staining by mason’s trichrome and hematoxylin/eosin staining. Alkaline phosphatase enzyme activity of implants was analyzed by staining sections with NBT/BCIP colorimetric substrate (Sigma-Aldrich, St. Louis, MO, USA).

### 4.5. Long Bone Micro Computed Tomography

Tibial bones from *Alpl*^−/−^ and *Alpl*^+/+^ day-17 mice were scanned by micro computed tomography (micro CT) using a Scanco μCT 100 micro-computed tomography system at a 18 µm isotropic voxel resolution and associated software. Trabecular region of interest used was 10% of total bone length from the end of the proximal growth plate. Cortical region of interest used was 10% of total bone length from the mid-diaphysis.

### 4.6. BMSC Cell Culture and Assay

For colony forming unit (CFU) assays, isolated cells were directly plated at 5 × 10^5^ cells per well into 12 well plates (passage 0 cells). For general colony forming units (CFU-F), cells were cultured in DMEM containing 20% FBS with media changes every 12 h for 2 days, then every 3 days. After 8 days of culture, the cells were fixed in methanol and stained with crystal violet. For colony forming adipocyte units (CFU-Ad), cells were cultured as for CFU-F and then cultured in adipocyte induction media (DMEM media containing 0.5 mM IBMX, 1μM dexamethasone, 100 μg/mL insulin, 10 μM troglitazone and 10% FBS and P/S) for 3 days followed by adipocyte maintenance media (DMEM media containing 100 μg/mL insulin, 10% FBS and P/S) for 3 days. Cells were then fixed and stained with Oil Red O (Abcam, Cambridge, MA, USA). For colony forming alkaline phosphatase positive units (CFU-AP), cells were cultured as for CFU-F and then in αMEM media containing 50 μg/mL ascorbate, 10% FBS, and P/S for 6 days. Cells were fixed and then stained for alkaline phosphatase activity using the colorimetric substrate, NBT/ BCIP (Sigma-Aldrich, St. Louis, MO, USA). After staining, plates were scanned and images were quantified using Image J. Comparison between genotypes was performed using the Student’s *t*-test (*n* = 3 per genotype per experiment).

### 4.7. BMSC Quantitative Real Time PCR

Passage 2 cells were induced to differentiate into osteoblasts by culturing in αMEM containing 50 μg/mL ascorbate, 10% FBS, and P/S for 6 days. Passage 2 cells were induced to differentiate into adipocytes by culturing in adipocyte induction media (0.5 mM 3-Isobutyl-1-methylxanthine, Sigma I5879; 1 µM Dexamethasone, Sigma D1756; 100 µg/mL insulin, Sigma I0516; 10 µM Troglitazone, Sigma T2573 in 10% FBS DMEM with 1% P/S) for three days followed by culturing in adipocyte maintenance media (100 µg/mL insulin in 10% FBS DMEM with 1% P/S) for three more days. RNA was isolated using Trizol reagent (Thermo Fisher, Waltham, MA, USA) following manufacturer protocol. Gene specific mRNA levels were assayed by reverse transcription followed by real time PCR. Real time PCR was performed using Taqman primer sets and Taqman Universal PCR Master Mix (Thermo Fisher Scientific, Waltham, MA, USA) on a 7500 Real Time PCR System (Thermo Fisher Scientific, Waltham, MA, USA) and quantified by comparison to a standard curve. The osteoblast genes included osteocalcin (OCN; bone gamma-carboxyglutamic acid-containing protein, *bglap*), BSP (bone sialoprotein, *ibsp*), collagen, type 1, alpha 1 (*col1a1*), and osterix (OSX, *sp7*). The adipocyte genes included *pparg* (peroxisome proliferator-activated receptor gamma), *fabp4* (fatty acid binding protein 4), adipsin (complement factor D), *CFD*, and *adipoq* (adipocyte specific protein). mRNA levels are reported after normalization to glyceraldehyde 3-phosphate dehydrogenase (*gapdh*) mRNA levels.

### 4.8. Strength and Motor Coordination Tests

For the grip strength test, eight weights were used: 0.15, 0.45, 1, 1.5, 2, 4.5, 6, and 10 g. For each test, the mouse was held by the middle/base of the tail and allowed to grasp the lightest weight which was lying on a flat laboratory benchtop. After the mouse grasped the weight with its forepaws, the mouse was raised until the weight was clear of the bench. A stopwatch was used to record the time. A hold of three seconds was the criterion. If the mouse dropped the weight in less than 3 sec, the mouse was allowed to try once again. If the mouse failed three times, the trial was terminated, and the mouse was assigned the lesser maximal weight. If the mouse held the weight for 3 sec, then the next heavier weight was tested. The test was run until the maximal weight was achieved. A final total score was calculated as the maximal weight, and compared between *Alpl*^−/−^ and *Alpl*^+/+^ mice. To compensate the difference in the body weight, the ratio of maximal weight/body weight was also calculated for each mouse and compared between *Alpl*^−/−^ and *Alpl*^+/+^ mice.

For Kondziela’s inverted screen test, the inverted screen was a 48 cm square of wire mesh consisting of 15 mm squares of 1 mm diameter wire. The mouse was placed in the center of the wire mesh screen. The screen was inverted and held 50 cm above a cushioned flat benchtop. A stopwatch was used to record the time when the mouse fell off, or the mouse was removed when the criterion time of 60 sec was reached. A final total score was calculated as the fall-off time, and compared between *Alpl*^−/−^ and *Alpl*^+/+^ mice.

For the horizontal bar test, the horizontal bar (4 mm in diameter and 38 cm in length) was held 50 cm above a cushioned flat benchtop. The mouse was held by its tail and aligned perpendicularly to the bar. The mouse was rapidly raised. Once its forepaws grasped the horizontal bar at the central point, its tail was released. A stopwatch was used to record the time of a fall from the bar. Maximum test time (cut-off time) was 30 s. A final total score was calculated as the fall-off time, and compared between *Alpl*^−/−^ and *Alpl*^+/+^ mice.

### 4.9. TNAP Deficient Cranial Osteoprogenitor and Muscle Progenitor Cells

MC3T3E1 cells were generously provided by Dr. Renny Franceschi (University of Michigan, Ann Arbor, MI, USA). Sol8 cells were acquired from the American Type Culture Collection (ATCC, Manassas, VA, USA). MC3T3E1 murine cranial osteoprogenitor cells and Sol8 murine skeletal muscle progenitor cells were transduced with lentiviral particles expressing a puromycin resistance gene and *Alpl* specific shRNA (Sigma Mission) or non-target shRNA (Sigma Mission, SHC002V) in the presence of 8 μg/mL hexadimethrine bromide. Puromycin-resistant colonies were expanded, confirmed for expression of TNAP and utilized for experiments (Supplementary Figure S3) [57].

Primary osteoprogenitor cells were isolated from the cranium of *Alpl*^+/+^ and *Alpl*^−/−^ mice by sequential collagenase digestion, as previously described [58,59,60]. Briefly, bones were rinsed with media then serially digested in a solution containing 2 mg/mL collagenase P and 2.5 mg/mL trypsin. Cells from the third digestion were used for experimentation as earlier digestion isolations contain cell from residual soft tissues and later digestion isolations contain osteocytes. Passage 3 cells were used for experiments.

### 4.10. Proliferation and MTT Assays

To assay for cell proliferation and MTT reduction, cells were seeded at an optimal density for each individual cell type in 6 well plates (MC3T3E1: 2.5 × 10^3^/well; primary cranial cells: 1 × 10^5^/well; BMSC: 2.5 × 10^3^/well; Sol8: 2.5 × 10^3^/well), and grown in aMEM media containing 10% FBS plus P/S for indicated number of days. Cells were stained with trypan blue and counted in sextuplet at each time point. Cellular metabolic activity was initially monitored by measuring the reduction of MTT. In brief, cells were plated in 96-well plates and cultured in Dulbecco’s modified Eagle’s medium containing 10% fetal bovine serum for indicated growth periods. The medium was replaced with 1 μg/mL MTT in phosphate-buffered saline (pH 7.4), followed by incubation at 37 °C for 3 h. MTT solution was removed, and cells were incubated in DMSO at 37 °C for 1 h. Reduction of MTT was quantified by measuring absorbance at 570 nanometers using a multi-well spectrophotometer.

### 4.11. Seahorse Agilent Seahorse XF Cell Mito Stress Test

To evaluate the changes in mitochondrial function and cell metabolism, cells underwent Seahorse Agilent Seahorse XFe Cell Mito Stress test (Agilent, Santa Clara, CA, USA) to measure changes in mitochondrial function via oxygen consumption rate (OCR). Agilent Seahorse XFe cell mito stress test was performed according to the manufacturer’s instructions. In brief, cells were seeded at the optimized cell density for each of the different cell lines in Seahorse XFe96 microplates and incubated at 37 °C/5% CO_2_ for 24 h. On the day of assay, the cell culture growth medium in the cell culture microplate was replaced with pre-warmed (37 °C) assay medium (XF DMEM, 1 mM Pyruvate, 2 mM Glutamine, and 10 mM glucose). The cell culture microplate was incubated in a non-CO2 incubator at 37 °C for 1 h prior to the assay to allow media temperature and pH to reach equilibrium. The modulating agents (oligomycin, FCCP, and antimycin A + rotenone) were prepared in assay medium and injected into the injection ports. The OCR was measured and analyzed using the Seahorse XFe Mito Stress Test Report Generator. At the end of the assay, cells were stained with crystal violet. The number of stained cells was counted and used for normalization.

### 4.12. ATP Measurements

Intracellular ATP levels were measured using a commercially available kit (ab83355, abcam, Cambridge, MA, USA), following manufacturer’s recommendations. Sol8 cells were seeded at 1 × 10^6^/well and MC3T3E1 cells were seeded at 2.5 × 105/well per well of 6-well plate. Briefly, cells were harvested, resuspended, deproteinized, and mixed with reaction reagent. The collected samples measured at 535/587 (SpectraMax i3x, Molecular devices, San Jose, CA, USA).

### 4.13. Immunofluorescent Staining for TNAP and Mitochondria 

Undifferentiated MC3T3E1 cranial bone progenitor cells, bone marrow stromal cells and Sol8 skeletal muscle progenitor cells were stained by immunofluorescence using Alexa Fluor 488 phalloidin for detection of F-actin (Thermo Fisher, Waltham, MA, USA), a rabbit anti-ALPL primary antibody (ab65834; Abcam, Cambridge, MA, USA), goat anti-rabbit Alexa Fluor-555 secondary antibody (Invitrogen, Carlsbad, CA, USA), and DAPI (4′,6-diamidino-2-phenylindole) nuclear stain to initially establish cellular localization of TNAP. For potential detection of co-localization of TNAP with mitochondria, cells were treated with 100 nM MitoTracker Red CMXRos (Invitrogen, Carlsbad, CA, USA) for 45 min. The mitochondrial dye was removed, and the cells were fixed. Cells were then stained with a monoclonal rat anti-ALPL primary antibody (MAB29091; R&D Systems, Minneapolis, MN, USA), donkey anti-rat Alexa Fluor-488 secondary antibody (Invitrogen, Carlsbad, CA, USA), and DAPI nuclear stain. Immunofluorescent staining was imaged using a Nikon Eclipse Ti microscope.

### 4.14. Statistics

*In vitro* data were assessed using Student’s *t*-test. For in vivo assays, data were tested for normality using the D’Agostino’s K-squared test. Student’s t-test was used for normal data, and Mann–Whitney U test was used for non-normal data analysis. A *p*-value less than 0.05 was considered statistically significant.

## 5. Conclusions

In this study, we investigated the need for TNAP in bone formation, mineralization, and mitochondrial function. We found that TNAP deficiency decreased trabecular bone volume fraction and trabeculation in addition to decreased mineralization, and interpret these results to mean that TNAP is essential for osteogenesis in addition to the mineralization of trabecular bone. We also showed for the first time that *Alpl*^−/−^ mice (global TNAP knockout) exhibit muscle and motor coordination deficiencies that are similar to those found in individuals with hypophosphasia/TNAP deficiency. Subsequent studies showed diminished proliferation, with mitochondrial hyperfunction and significantly increased ATP levels in TNAP deficient bone and muscle progenitor cells. We also found that TNAP is expressed in a peri-nuclear intracellular pattern in these cells. Together, our results indicate that TNAP functions inside bone and muscle progenitor cells to influence mitochondrial respiration and ATP production. Future studies are required to establish mechanisms by which TNAP influences mitochondrial function, to determine the extent to which TNAP induced mitochondrial hyper respiration causes musculoskeletal defects seen in *Alpl*^−/−^ mice and individuals with hypophosphatasia, and establish that modulation of TNAP can alter mitochondrial respiration in vivo.

## Figures and Tables

**Table 1:** TNAP deficiency alters mitochondrial and non-mitochondrial metabolic activity.

Cell Type	Geno Type	Basal Respiration	ATP Production	Proton Leak	Maximal Respiration	Space Capacity	Non-Mito Oxygen Consumption
MC3T3E1 Cranial Osteoprogenitor Cell Line	NT shRNA	2.4±0.3	1.9±0.3	0.54±0.08	6.0±1.2	3.6±0.90	0.9±0.4
	*Alpl* shRNA	4.8±0.6 **	3.8±0.6 *	0.99±0.03 **	11.5±2.2 *	6.7±1.7	1.6±0.5
Primary Cranial Osteoprogenitor Cells	*Alpl* ^+/+^	10.8±0.6	9.3±0.5	1.40±0.20	28.8±5.5	18.0±5.5	4.9±0.5
	*Alpl* ^−/−^	12.6±1.0 **	10.3±0.5 *	2.27±0.56 *	33.6±5.9	21.0±6.1	6.3±0.6 **
Primary Bone Marrow Stromal Cells	*Alpl* ^+/+^	6.2±0.3	5.2±0.4	1.05±0.48	12.0±1.7	5.8±1.6	3.0±0.9
	*Alpl* ^−/−^	9.4±0.8 *	7.5±0.7 **	1.83±0.33	16.4±1.2 *	7.1±1.6	2.1±0.4
Sol8 Skeletal Muscle Cell Line	NT shRNA	33.9±4.6	27.1±3.7	6.83±1.63	72.1±13.5	38.2±10.0	11.9±4.3
	*Alpl* shRNA	50.7±6.3 **	40.6±.8 **	10.14±1.58 **	152.5±21.2 ***	101.8±16.9 ***	18.4±2.7 *

Results shown are means ± standard error. *n* = 3 per genotype. * *p* < 0.05, ** *p* < 0.01, *** *p* < 0.005 statistical significance between genotypes. Non-Mito = non-mitochondrial; NT = non-target/control.

## Data Availability

Not applicable.

## Supplementary Materials

The following are available online at https://www.mdpi.com/1422-0067/22/3/1140/s1, Figure S1: Cortical and trabecular bone parameters of donor Alpl+/+ and Alpl-/- tibias, Figure S2: Skeletal staining demonstrates diminished mineralization and size of Alpl+/+ and Alpl-/- mouse long bones. Tibias were stained with alizarin red for bone mineral and alcian blue for cartilage. Note shorter bone length and reduced alizarin staining in Alpl-/- bones, Figure S3: Real time PCR demonstrates that Sol8 cells stably transduced with shRNA for TNAP express significantly less Alpl (TNAP) mRNA than Sol8 cells stably transduced with non-target shRNA. NT = non-target.

## Abbreviations

Alpl	Gene encoding Alkaline Phosphatase, Liver/Bone/Kidney
TNAP	Tissue Nonspecific Alkaline Phosphatase Isozyme
HPP	Hypophosphatasia
ATP	Nucleotide Adenosine Triphosphate
P/S	Penicillin/Streptomycin
FBS	Fetal Bovine Serum
AdI/M	Adipocyte induction and maintenance media
Asc	Ascorbate
BMSC	Bone marrow stromal cell
CFU	Colony forming units
CV	Crystal violet
OCN	Osteocalcin; bone gamma-carboxyglutamic acid-containing protein (Bglap)
BSP	Bone Sialoprotein; Integrin Binding Sialoprotein (Ibsp)
Col1a1	Collagen, type I, alpha 1
OSX	Osterix; Sp family member 7 (Sp7)
Pparg	Peroxisome proliferator-activated receptor gamma,
Fabp4	fatty acid binding protein 4
Adipsin	Complement factor D (CFD)
AdipoQ	Adipocyte specific protein
GAPDH	Glyceraldehyde 3-phosphate dehydrogenase
FCCP	Carbonyl cyanide-4 (trifluoromethoxy) phenylhydrazone
OCR	Oxygen consumption rate
MC3TE1	Cranial osteoprogenitor cell line
Sol8	Skeletal muscle progenitor cell line
Nano CT	Nano computed tomography
Micro CT	Micro computed tomography
