# Supplementary material for: Tissue Nonspecific Alkaline Phosphatase Function in Bone and Muscle Progenitor Cells: Control of Mitochondrial Respiration and ATP Production
Source: Int J Mol Sci. 2021 Jan 24;22(3):1140. doi: 10.3390/ijms22031140 (PMC7865776; doi:10.3390/ijms22031140)

Supplementary Figure 1. Cortical and trabecular bone parameters of donor *Alpl*$^{+/+}$ and *Alpl*$^{-/-}$ tibias.

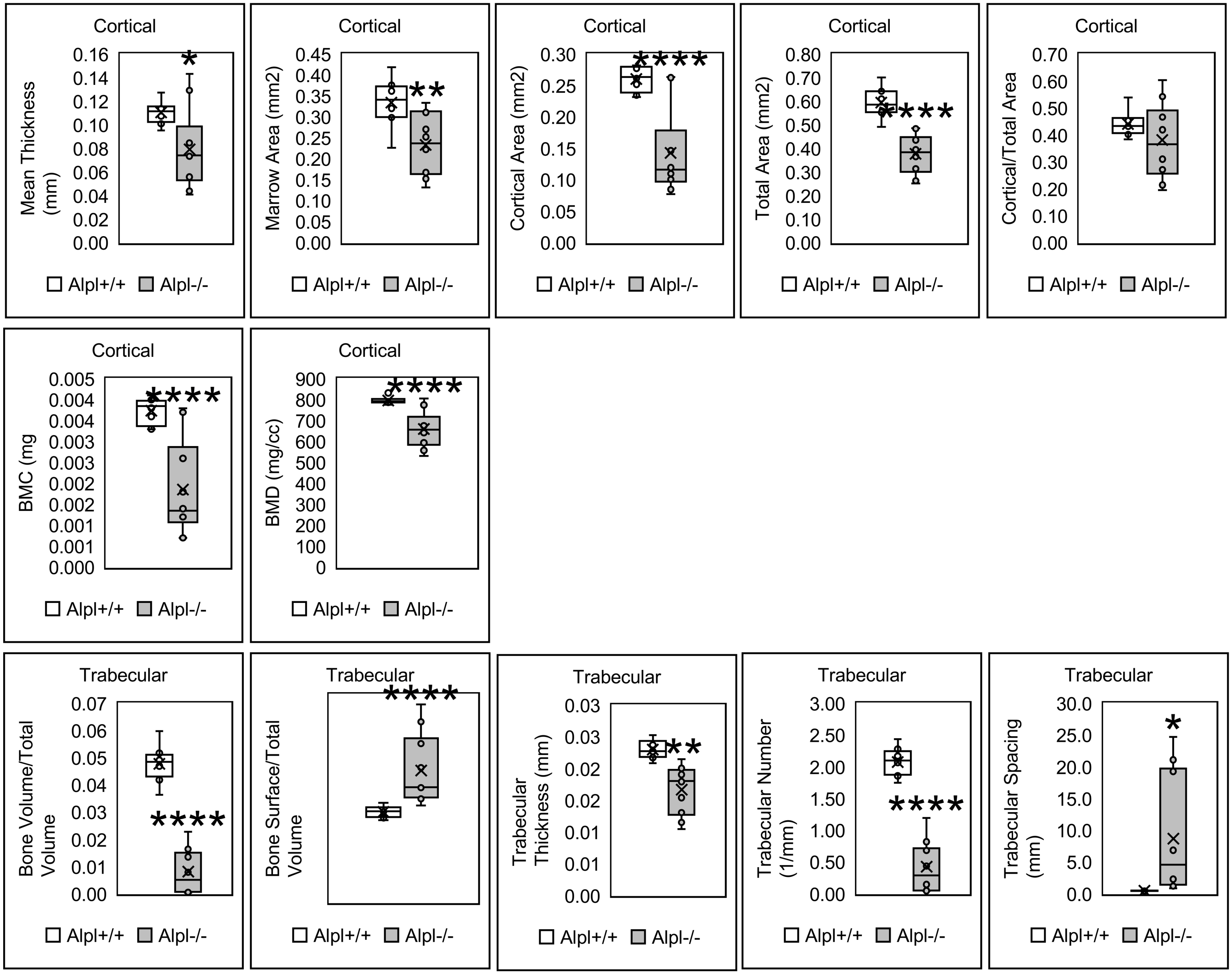

*p value < 0.05 between genotypes, ** p value < 0.01 between genotypes, *** p value < 0.005 between genotypes, **** p value < 0.001 between genotypes

Supplementary Figure 2. Skeletal staining demonstrates diminished mineralization and size of *Alpl*$^{+/+}$ and *Alpl*$^{-/-}$ mouse long bones. Tibias were stained with alizarin red for bone mineral and alcian blue for cartilage. Note shorter bone length and reduced alizarin staining in *Alpl*$^{-/-}$ bones.

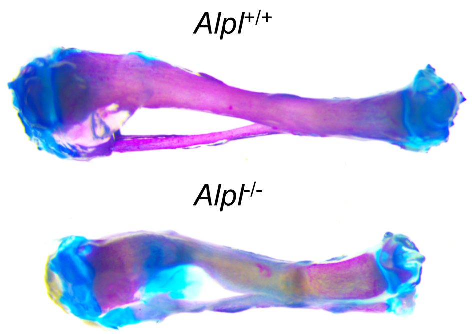

Supplementary Figure 3. Real time PCR demonstrates that Sol8 cells stably transduced with shRNA for TNAP express significantly less Alpl (TNAP) mRNA than Sol8 cells stably transduced with non-target shRNA. NT = non-target.

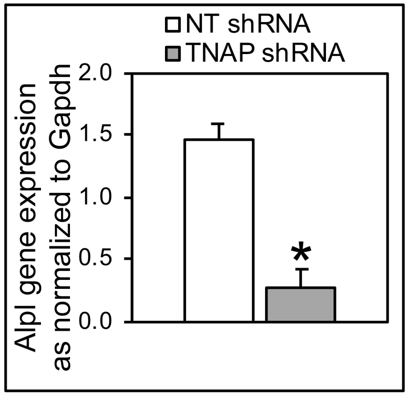

Supplement: Supplementary file 1 [file ijms-22-01140-s001.pdf]
